# Antibodies Against Modified NS1 Wing Domain Peptide Protect Against Dengue Virus Infection

**DOI:** 10.1038/s41598-017-07308-3

**Published:** 2017-08-01

**Authors:** Yen-Chung Lai, Yung-Chun Chuang, Ching-Chuan Liu, Tzong-Shiann Ho, Yee-Shin Lin, Robert Anderson, Trai-Ming Yeh

**Affiliations:** 1 The Institute of Basic Medical Sciences, College of Medicine, National Cheng Kung University, Tainan, Taiwan; 2 Department of Medical Laboratory Science and Biotechnology, College of Medicine, National Cheng Kung University, Tainan, Taiwan; 3 Department of Pediatrics, College of Medicine, National Cheng Kung University, Tainan, Taiwan; 4 Department of Microbiology and Immunology, College of Medicine, National Cheng Kung University, Tainan, Taiwan; 5 Departments of Microbiology & Immunology and Pediatrics, and Canadian Center for Vaccinology, Dalhousie University, Halifax, Nova Scotia Canada

## Abstract

Dengue is the most common mosquito-transmitted viral infection for which an improved vaccine is still needed. Although nonstructural protein-1 (NS1) immunization can protect mice against dengue infection, molecular mimicry between NS1 and host proteins makes NS1-based vaccines challenging to develop. Based on the epitope recognized by the anti-NS1 monoclonal Ab (mAb) 33D2 which recognizes a conserved NS1 wing domain (NS1-WD) region but not host proteins, we synthesized a modified NS1-WD peptide to immunize mice. We found that both mAb 33D2 and modified NS1-WD peptide immune sera could induce complement-dependent lysis of dengue-infected but not un-infected cells *in vitro*. Furthermore, either active immunization with the modified NS1-WD peptide or passive transfer of mAb 33D2 efficiently protected mice against all serotypes of dengue virus infection. More importantly, dengue patients with more antibodies recognized the modified NS1-WD peptide had less severe disease. Thus, the modified NS1-WD peptide is a promising dengue vaccine candidate.

## Introduction

Dengue virus (DENV) infection is the most widespread arthropod-borne viral disease, responsible for 390 million infections annually, mainly in tropical and sub-tropical countries^1^. The most common form of DENV disease is dengue fever (DF), which is self-limiting with symptoms such as headache, fever, and skin rash; however, a relatively small proportion of patients may progress to life-threatening dengue hemorrhagic fever (DHF) or dengue shock syndrome (DSS)^2, 3^. According to the most recent World Health Organization classification of dengue severity, which includes dengue with or without warning signs and severe dengue^4^, the main characteristics of DHF/DSS or severe dengue are abnormal hemostasis and plasma leakage. Unfortunately, due to the complicated immunopathogenic mechanisms underlying dengue disease, no satisfactory antiviral drugs or vaccine are available to treat or prevent DHF/DSS development^5–8^.

Dengue virus (DENV) is a single-stranded, positive-sense RNA flavivirus that has three structural proteins, envelope (E), matrix (M) and capsid (C) proteins, as well as seven nonstructural (NS) proteins^9^. Based on antigenic differences in the E protein, there are four serotypes of DENV. Among the seven NS proteins, NS1 is the only one that is expressed on the surface of DENV-infected cells as a dimer^10, 11^ and is also secreted as a hexamer in the circulation^12, 13^. DENV NS1 is a glycoprotein containing 352 amino acid residues. Crystal structure analysis has revealed that each NS1 monomer is arranged into three distinct domains: the wing domain (WD), the hydrophobic β-roll dimerization domain, and the central β-ladder domain, which is formed by the C-terminal half of NS1 (amino acids 181 to 352)^14, 15^. The secreted NS1 (sNS1) hexamer is composed of three dimers, which form a detergent-sensitive hydrophobic central cavity that carries lipid cargo^16, 17^. The serum levels of sNS1 can reach as high as 50 μg/ml in the acute stage of DHF/DSS infection^18^. Clinical evidence also indicates that levels of NS1 in dengue patient serum correlate with disease severity^19, 20^. The sNS1 may bind to cell membranes via interactions with heparan sulfate and chondroitin sulfate^21^. sNS1 can also interact with prothrombin to interrupt the coagulation cascade^22^. In addition, sNS1 can activate complement to elicit complement-dependent cytotoxicity in endothelial cells or to evade innate immune defense mechanisms^23, 24^. Recently, it has been shown that sNS1 can enhance vascular permeability, either via binding to endothelial cells or activating cytokine release from peripheral mononuclear cells thus possibly contributing to DHF^25, 26^. As a result, NS1 may act as a viral toxin and may also be considered a novel therapeutic and vaccine target.

Previous studies have shown that antibodies (Abs) against NS1 can directly target membrane-bound NS1 and cause complement-dependent lysis of DENV-infected cells *in vitro* ^27^. Passive transfer of anti-DENV NS1 Abs or active immunization with NS1 can protect mice from DENV challenge^25, 27–29^. However, several Abs against NS1 show molecular mimicry with host proteins and can cross-react with platelets, endothelial cells and coagulation factors, causing platelet dysfunction, endothelial cell damage and hemorrhage^22, 30–32^. These factors hinder the development of a potential NS1-based vaccine. As most of the cross-reactive Abs recognize the C-terminal region of NS1, C-terminal truncated NS1 or C-terminal modified NS1 have been used as potentially safer vaccine candidates^27, 33^. In addition to these dominant C-terminal epitopes, there are other regions of NS1 that can induce host cross-reactive Abs^34, 35^. Therefore, further epitope engineering is necessary for NS1-based vaccine design^5, 15^.

Based on structural studies of NS1, the WD disordered loop is a major surface exposed antigenic site that is also a highly conserved region among all four DENV serotypes. However, it has also been reported that monoclonal antibodies (mAbs) specific for NS1-WD can cross-react with the LYRIC protein expressed on human endothelial cells^34^. In this study, we successfully identified a unique anti-NS1 mAb 33D2 that can recognize the NS1-WD disordered loop but not LYRIC. Based on epitope mapping analysis of mAb 33D2, we synthesized a peptide of a modified NS1-WD sequence to immunize mice. Our results demonstrated that Abs generated against the modified NS1-WD peptide did not cross-react with host proteins but could inhibit DENV infection both *in vitro* and *in vivo*. Furthermore, active immunization with the modified NS1-WD peptide could provide protection against DENV-induced hemorrhage, coagulopathy, viremia, and mortality in mice. Importantly, clinical analyses revealed that higher sera levels of Abs recognized the modified NS1-WD peptide correlated with lower disease severity in dengue patients. Thus, our results suggest that this modified NS1-WD peptide may represent a new preventative DENV vaccine candidate.

## Results

### Dengue virus NS1-WD mAb 19–5 but not 33D2 cross-reacts with the LYRIC protein of human endothelial cells

**Figure 1:**
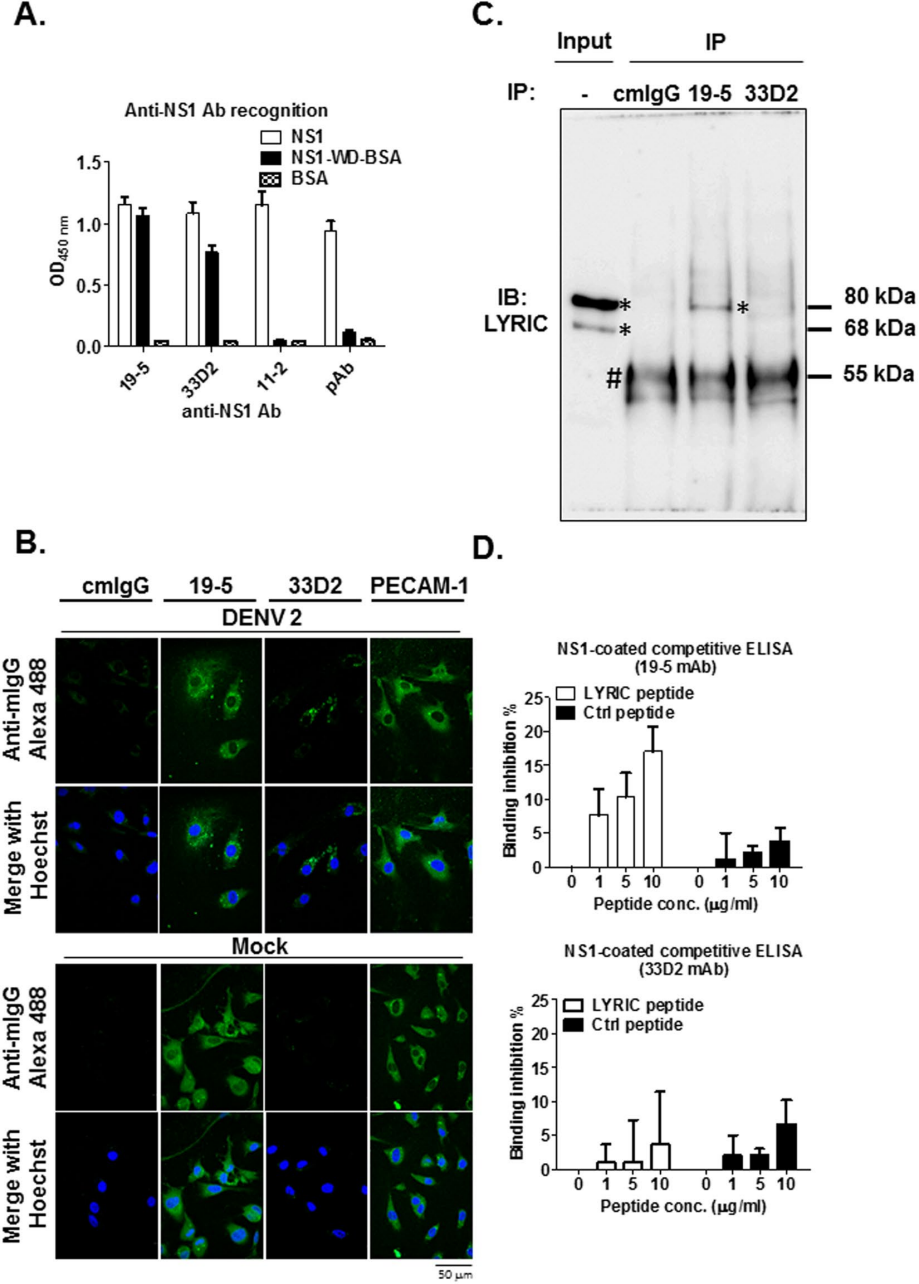
Dengue virus NS1-WD mAb 19-5 but not 33D2 cross-reacts with the LYRIC protein on human endothelial cells. **(A)** The binding of anti-NS1 mAbs 19-5, 33D2, 11-2 or pAb (1 μg/ml) to NS1, NS1-WD-conjugated BSA and BSA (2 μg/ml) was analyzed by ELISA. **(B)** HUVECs were infected with DENV 2 (moi = 10) or mock-infected for 48 h. Cells were stained with cmIgG, anti-NS1 Abs (19-5 and 33D2), or anti-PECAM-1 and analyzed by IIF surface staining, as described in the Methods. CmIgG and Anti-PECAM-1 served as negative and positive controls of endothelial cell staining, respectively. The scale bar indicated 50 μm. **(C)** Co-immunoprecipitation (IP) of HUVECs lysates with cmIgG, anti-NS1 mAb 19-5 or 33D2 followed by full-length immunoblotting with anti-LYRIC Ab. *Indicates LYRIC protein; ^#^indicates heavy chain of antibodies. **(D)** Different concentrations of LYRIC peptide were pre-incubated with anti-NS1 mAbs 19-5, 33D2, or pAb (0.1 μg/ml) and incubated in NS1 (2 μg/ml)-coated ELISA plates. Bound antibodies were detected as described in the Methods. The binding inhibition (%) represents the inhibitory percentage of pre-incubated peptides to antibodies compared to no peptide pre-incubation. All data are presented as the mean ± S.D. from at least three independent experiments.

To identify mAbs that recognize NS1-WD, we screened mAbs from insect cell-derived DENV NS1 immunized mice against the NS1-WD peptide (a.a.109–122). Two NS1-WD specific mAbs, 19–5 and 33D2, which recognized both full-length DENV serotype 2 (DENV 2) NS1 protein and NS1-WD peptide, were found (Fig. 1A). Both of them recognized all four serotypes of DENV-infected cells, as analyzed by indirect immunofluorescence (IIF) (Supplementary Fig. 1). However, we found that mAb 19-5 but not 33D2 could bind to the surface of uninfected human umbilical vein endothelial cells (HUVECs) as analyzed by IIF surface staining (Fig. 1B). To identify the target protein of mAb 19-5 on human endothelial cells, we used co-immunoprecipitation assays. As expected, mAb 19-5 but not 33D2 or isotype control mouse IgG (cmIgG) cross-reacted with the LYRIC protein of HUVECs (Fig. 1C). To further confirm the region recognized by mAb 19-5, we performed competitive binding assays. We found the binding of mAb 19-5 but not 33D2 to NS1 was inhibited by pre-incubation with various doses of synthetic LYRIC peptide but not a control peptide (nonspecific peptide) (Fig. 1D). Collectively, these data suggest that NS1 immunization indeed induced Abs, which recognized NS1-WD. Among them, mAb 19-5 but not mAb 33D2 showed cross reactivity with the HUVEC LYRIC protein.

### Abs against a modified NS1-WD peptide can recognize four serotypes of DENV-infected cells, but their cross-reactivity to HUVECs is reduced

**Figure 2:**
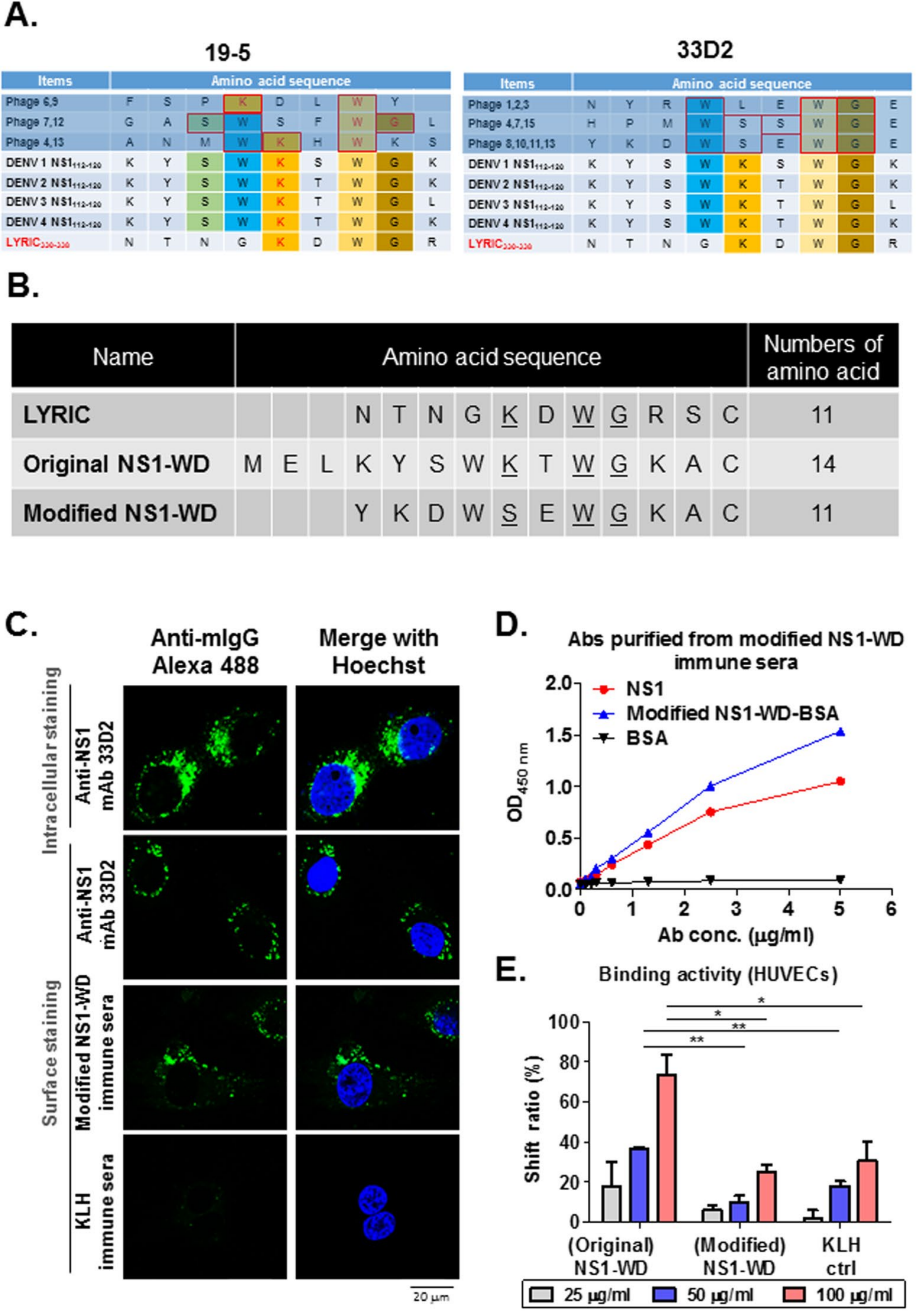
Antibodies against modified NS1-WD peptide recognize all four serotypes of DENV-infected cells and show reduced cross-reactivity to HUVECs. (**A**) Consensus sequence of mAb 19-5 and 33D2 phagetopes shared with LYRIC and NS1 were analyzed. The amino acids depicted with different color backgrounds are identical between the four serotypes of DENV NS1 and LYRIC. (**B**) The sequence of the modified NS1-WD peptide was compared to those of the original NS1-WD and LYRIC peptide (**C**) HUVECs were infected with DENV 2 (moi = 10) for 48 h. Cells were stained with anti-NS1 mAb 33D2, modified NS1-WD immune sera, or KLH immune sera (1:100 dilution) and analyzed by surface or intracellular IIF staining as described in the Methods. The scale bar indicates 20 μm. (**D**) The binding of purified pAbs from modified NS1-WD peptide-immunized mouse sera to NS1, modified NS1-WD peptide-conjugated BSA, or BSA was detected by ELISA. (**E**) The binding of different doses of purified pAbs from original NS1-WD, modified NS1-WD peptide immune sera or cmIg to HUVECs was analyzed by flow cytometry. The shift ratio (%) represents the percentage of binding compared with the control group (Anti-mouse IgG Alexa 488 only). All data are presented as the mean ± S.D. from at least three independent experiments. *P < 0.05, **P < 0.01, ***P < 0.001, ns indicates no significance.

To identify the precise target sequences recognized by anti-NS1 mAbs 19-5 and 33D2, we used 12-mer phage-displayed random peptide libraries. Three clusters of consensus sequences were found in mAb 19-5 and 33D2 phagetopes. Interestingly, alignment analysis revealed phagetopes of mAb 19-5 and 33D2 that matched the conserved sequence of all four serotypes of DENV NS1 (a.a. 114–119), while a slightly different sequence aligned to the LYRIC protein (a.a. 332–337). The epitope of mAb 33D2 contained no KXWG motif, which is critical to the cross reactivity with the LYRIC protein (Fig. 2A). In addition, to avoid any other pathogenic epitopes such as the ELK/KEL-type motifs in the NS1-WD region^36^, we not only modified the original NS1-WD sequence according to mAb 33D2 epitope mapping analysis, but we also deleted three of the N-terminal amino acids, resulting in a peptide consisting of 11 amino acids (Fig. 2B). Our data showed that modified NS1-WD peptide immune sera could still recognize NS1 on the cell surface of DENV 2-infected HUVECs, as analyzed by IIF (Fig. 2C), and all four serotypes of DENV-infected HuH-7 cells, as analyzed by flow cytometry (Supplementary Fig. 2). Furthermore, the polyclonal Abs purified from modified NS1-WD peptide-immunized mice (modified NS1-WD Abs) bound to NS1 in a dose-dependent manner (Fig. 2D). Subsequently, we examined the cross reactivity of modified NS1-WD Abs to HUVECs. Unlike the original NS1-WD peptide immune sera, the binding to HUVECs of Abs purified from modified NS1-WD peptide immune sera was similar to KLH immune sera (Fig. 2E, Supplementary Fig. 3). These results suggest that the modified NS1-WD peptide induced Abs that could recognize the four different serotypes of DENV NS1 but not HUVECs.

### Passive transfer of mAb 33D2 protects mice against DENV-elicited pathogenesis

**Figure 3:**
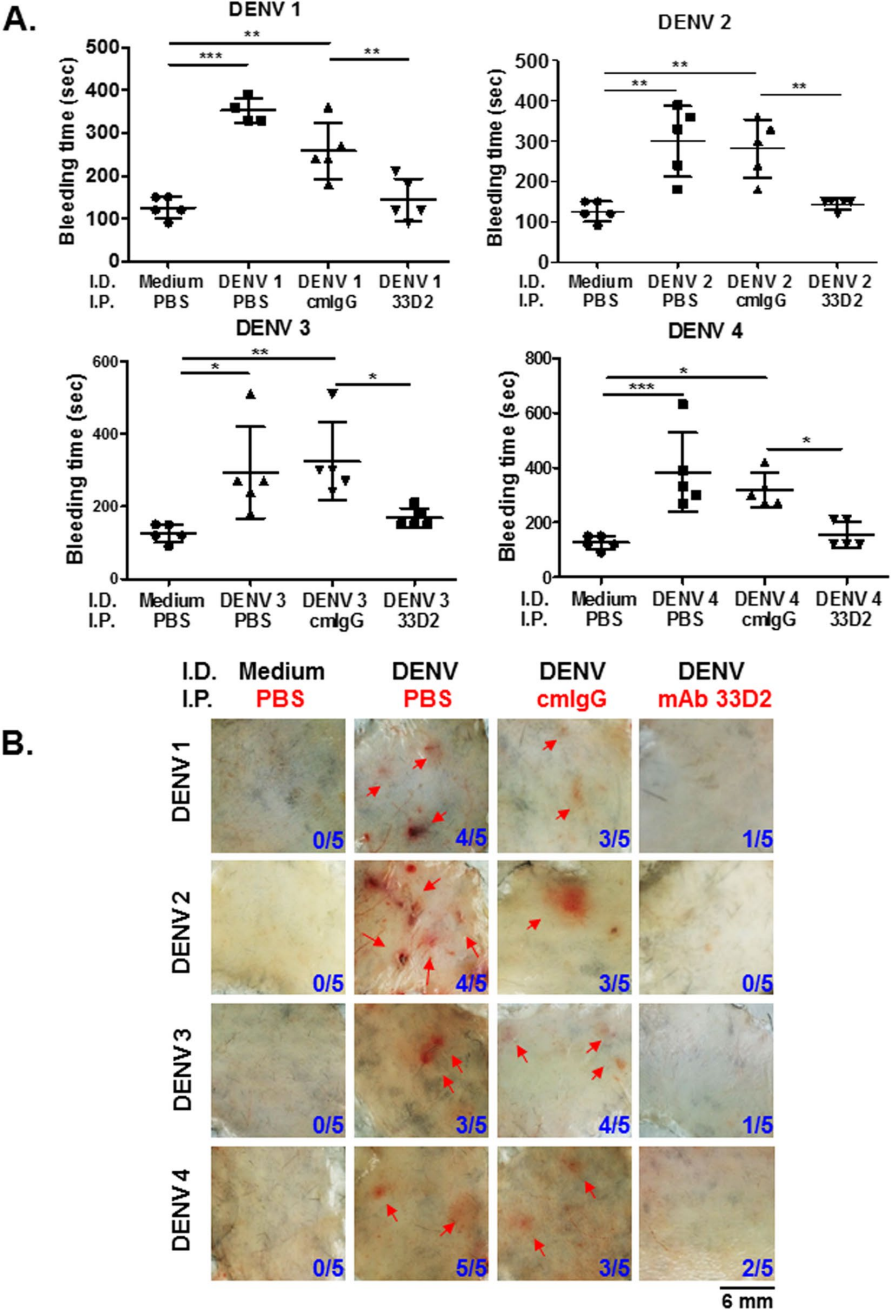
Passive transfer of mAb 33D2 protects mice against DENV–elicited hemorrhage and coagulopathy. Hemorrhagic mouse model was created as described in the Methods (N = 5 for each group). **(A)** The tail bleeding time was determined on day 3. **(B)** Fresh skin samples from mice were removed to observe local hemorrhage. The number of mice with hemorrhage/total number of mice inoculated in each group are indicated. Red arrows indicate local skin hemorrhage. The scale bar indicates 6 mm. *P < 0.05, **P < 0.01, ***P < 0.001, ns indicates no significance.

To test whether administration of mAb 33D2 can alleviate DENV-induced disease development *in vivo*, a previously established DENV-induced hemorrhagic C3H/HeN mouse model was used^27, 37^. As previously demonstrated, inoculation of mice with high titer DENV (2*10^8^ PFU/mouse) can produce pathogenic signs similar to dysfunction of platelets and hemorrhage in dengue patients, such as prolonged bleeding time and local hemorrhage in skin (Fig. 3). In addition, we found that secreted NS1 in DENV-inoculated but not inactivated DENV-inoculated mice was increased as measured by NS1-sandwich ELISA. The kinetics of secreted NS1 in sera of mice indicated that DENV replication peaked at 12 h post infection and then gradually declined to undetectable levels at 72 h post infection. (Supplementary Fig. 4). Subsequently, mice were inoculated with DENV intradermally. After the first 24 h of infection, 100 μg cmIgG and 33D2 mAb were administered intraperitoneally to test the therapeutic effect of the 33D2 mAb in mice. The results showed that prolonged bleeding time induced by all four serotypes of DENV was effectively shortened in mAb 33D2, but not in cmIgG-treated mice (Fig. 3A). Notably, DENV-elicited local skin hemorrhage, which was digitally quantified by ImageJ software analysis, was also blocked in mAb 33D2-treated mice (Fig. 3B, and Supplementary Fig. 5). In addition, because plasma leakage is another main characteristic of DHF/DSS, we evaluated the permeability change in our mouse model. We demonstrated that mAb 33D2 not only rescued the NS1-induced permeability increase *in vitro* but also attenuated DENV-induced vascular hyperpermeability in the lungs and skin *in vivo* (Supplementary Fig. 6). Collectively, these results indicate that passive transfer of mAb 33D2 effectively protected against DENV-induced disease development.

### Modified NS1-WD peptide immune sera provide protection against four serotypes of DENV infection via complement-dependent cytolysis of infected cells

**Figure 4:**
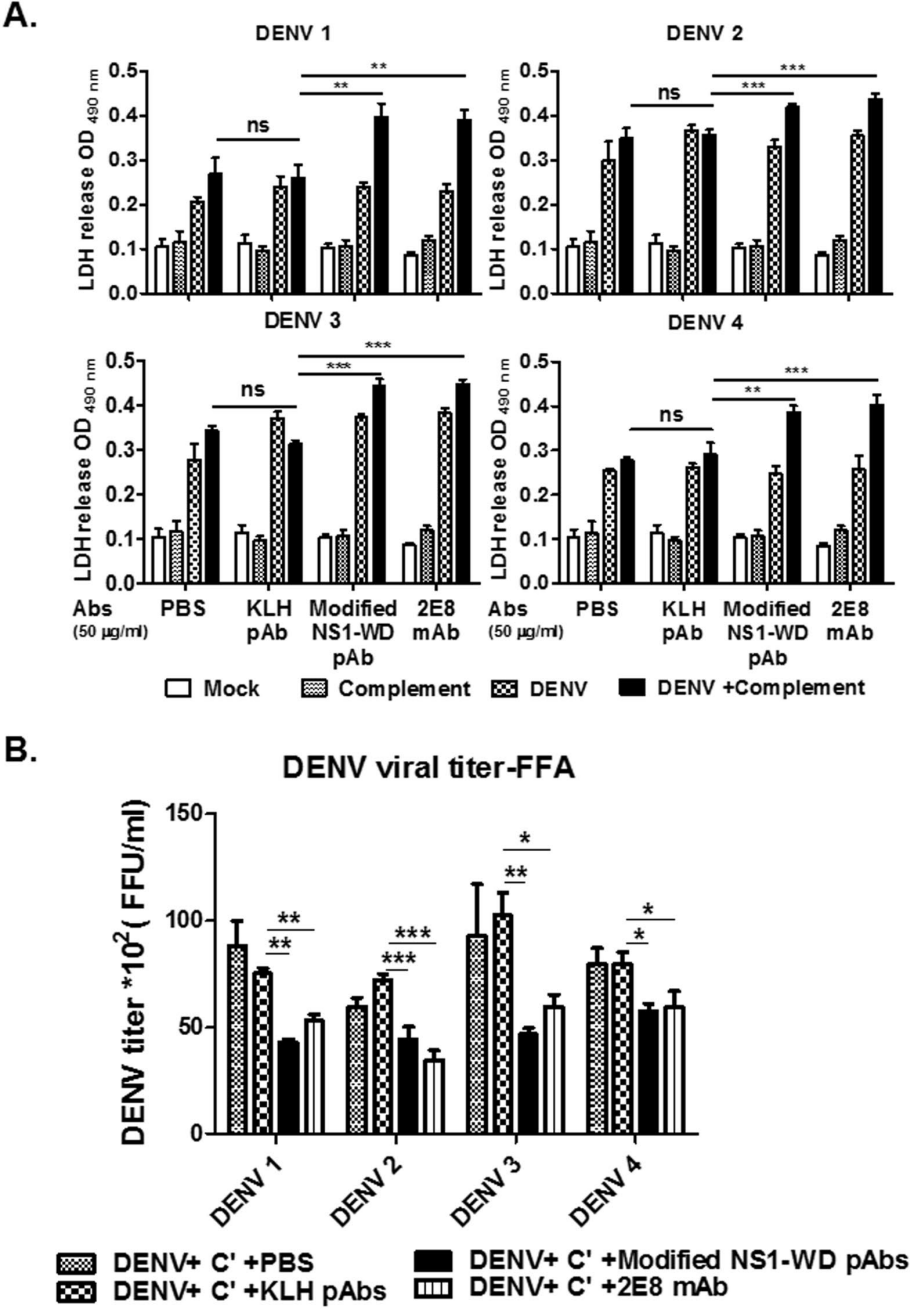
Antibodies against modified NS1-WD peptide reduce viral replication by complement-dependent cytolysis of DENV-infected cells. (**A**) HUVECs were infected with DENVs (serotypes 1–4, moi = 10) or mock infection for 48 h. After 48 h of infection, cells were incubated with either PBS, purified pAbs from KLH or modified NS1-WD-peptide immune sera (50 μg/ml) or anti-NS1 mAb 2E8 (50 μg/ml) for 1 h at 4 °C and incubated with or without complement (1:20) for 4 h at 37 °C. The release of lactate dehydrogenase (LDH) was analyzed as described in the Methods. Anti-NS1 Ab 2E8 served as a positive control. (**B**) HUVECs were infected with DENVs (serotypes 1–4, moi = 10) or mock-infected for 48 h. After 48 h of infection, cells were incubated with either PBS, purified pAbs from KLH or modified NS1-WD peptide-immunized mouse sera (50 μg/ml) or anti-NS1 mAb 2E8 (50 μg/ml) for 1 h at 4 °C and incubated with complement (**C’**) (1:20) for 4 h at 37 °C. After refilling with fresh medium for another 24 h, the infectious virus in supernatants was titrated by FFA as described in the Methods. All data are presented as the mean ± S.D. from at least three independent experiments. *P < 0.05, **P < 0.01, ***P < 0.001, ns indicates no significance.

Given that mAb 33D2 provides protection against DENV infection in mice by passive transfer, we tested whether active immunization with modified NS1-WD peptide can also protect mice from DENV infection. We first showed that modified NS1-WD Abs, such as a positive control anti-NS1 mAb 2E8 but not a negative control KLH Abs, could activate complement, inducing significant LDH release in DENV-infected but not uninfected HUVECs (Fig. 4A) and HuH-7 cells (Supplementary Fig. 7). The virus titers in the supernatants were further analyzed by fluorescent focus assay (FFA). In agreement with the LDH release assay results, the virus titers in all four different serotypes of DENV-infected cells treated with modified NS1-WD Abs were significantly reduced compared with cells treated with KLH pAbs or PBS (Fig. 4B). These results indicate that modified NS1-WD Abs could reduce virus replication by complement-mediated lysis of infected cells.

### Modified NS1-WD peptide immunization protects against DENV-elicited hemorrhage and coagulopathy in mice

**Figure 5:**
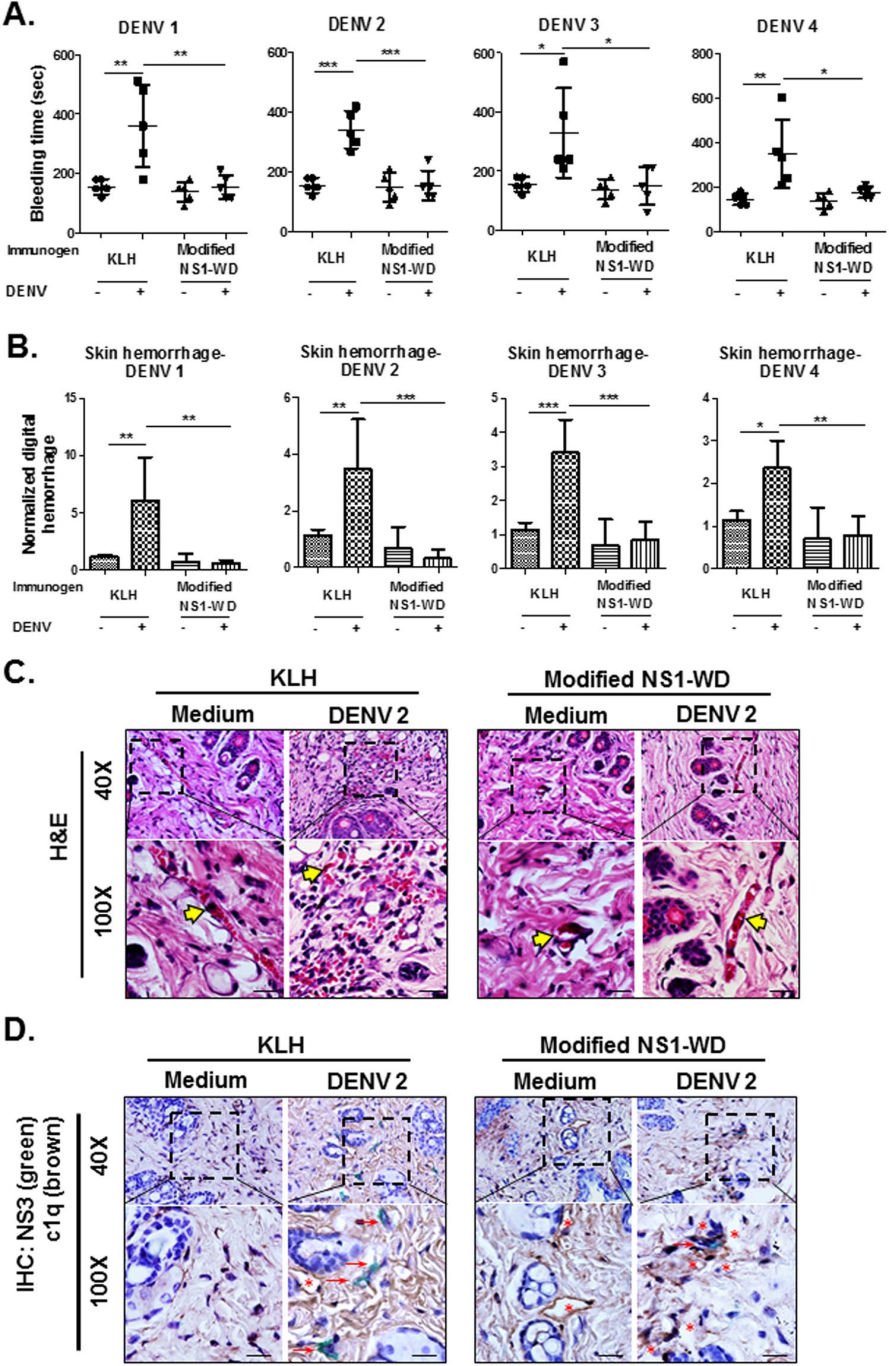
Active immunization of modified NS1-WD peptide protects mice against DENV–elicited hemorrhage and coagulopathy. Disease mouse model was created as described in the Methods (N = 5 for each group). **(A)** The tail bleeding time was determined on day 3. (**B)** The clinical score of hemorrhage was quantified and determined as digital hemorrhage severity by ImageJ. **(C)** H&E staining was performed to analyze local skin hemorrhage in the skin lesions. The yellow arrows indicate the intact blood vessels. The scale bar indicated 40 μm. **(D)** DENV NS3 expression (green staining) and complement c1q deposition (brown staining) were depicted in IHC double staining. Red arrows indicate local DENV NS3 expression, and red stars indicate c1q deposition. The scale bar indicates 40 μm. *P < 0.05, **P < 0.01, ***P < 0.001, ns indicates no significance.

Based on the promising therapeutic results demonstrated above, we further evaluated the possibility of using modified WD-NS1 peptide as a vaccine candidate against DENV infection. To compare the efficacy and safety of different NS1 protein epitopes, we immunized mice with either control carrier protein (KLH), modified NS1-WD peptide-conjugated KLH or C-terminal NS1 peptide (a.a.305–314, GKLITEWCCRC)-conjugated KLH, which contained a pathogenic epitope of NS1^32^. Subsequently, the antibody titers against NS1 were determined by ELISA before DENV challenge (Supplementary Fig. 8A). Three days after intradermal DENV inoculation, we found that DENV 2-induced prolonged bleeding time and hemorrhage were significantly attenuated in mice immunized with modified NS1-WD peptide but not with KLH (Supplementary Fig. 8B,C). In contrast, DENV 2-induced hemorrhage was even greater in mice immunized with C-terminal NS1 peptide (Supplementary Fig. 8C). Furthermore, prolonged bleeding and hemorrhage induced by other serotypes of DENV were also attenuated in mice immunized with modified NS1-WD peptide, but not with KLH (Fig. 5A,B and Supplementary Fig. 9). To examine the pathological changes, H&E staining of the skin dermis was performed which showed that DENV 2-elicited red blood cell extravasation was attenuated in mice immunized with modified NS1-WD peptide (Fig. 5C). Additionally, we also determined the reduction of DENV 2 NS3 expression, a marker of DENV replication. There was at least a two-fold decrease in the quantification of NS3 expression in the skin dermis of mice immunized with modified NS1-WD peptide (Supplementary Fig. 10). In addition, there was more deposition of complement c1q (DAB brown staining) surrounding DENV-infected cells (HRP green staining) in the skin dermis of mice immunized with modified NS1-WD peptide, as showed by immunohistochemistry staining (Fig. 5D). Taken together, these results indicate that modified NS1-WD Abs could reduce virus replication by complement-mediated lysis of DENV-infected cells both *in vitro* and *in vivo*.

### Both modified NS1-WD peptide specific Abs and mAb 33D2 protect mice from lethal DENV infection

**Figure 6:**
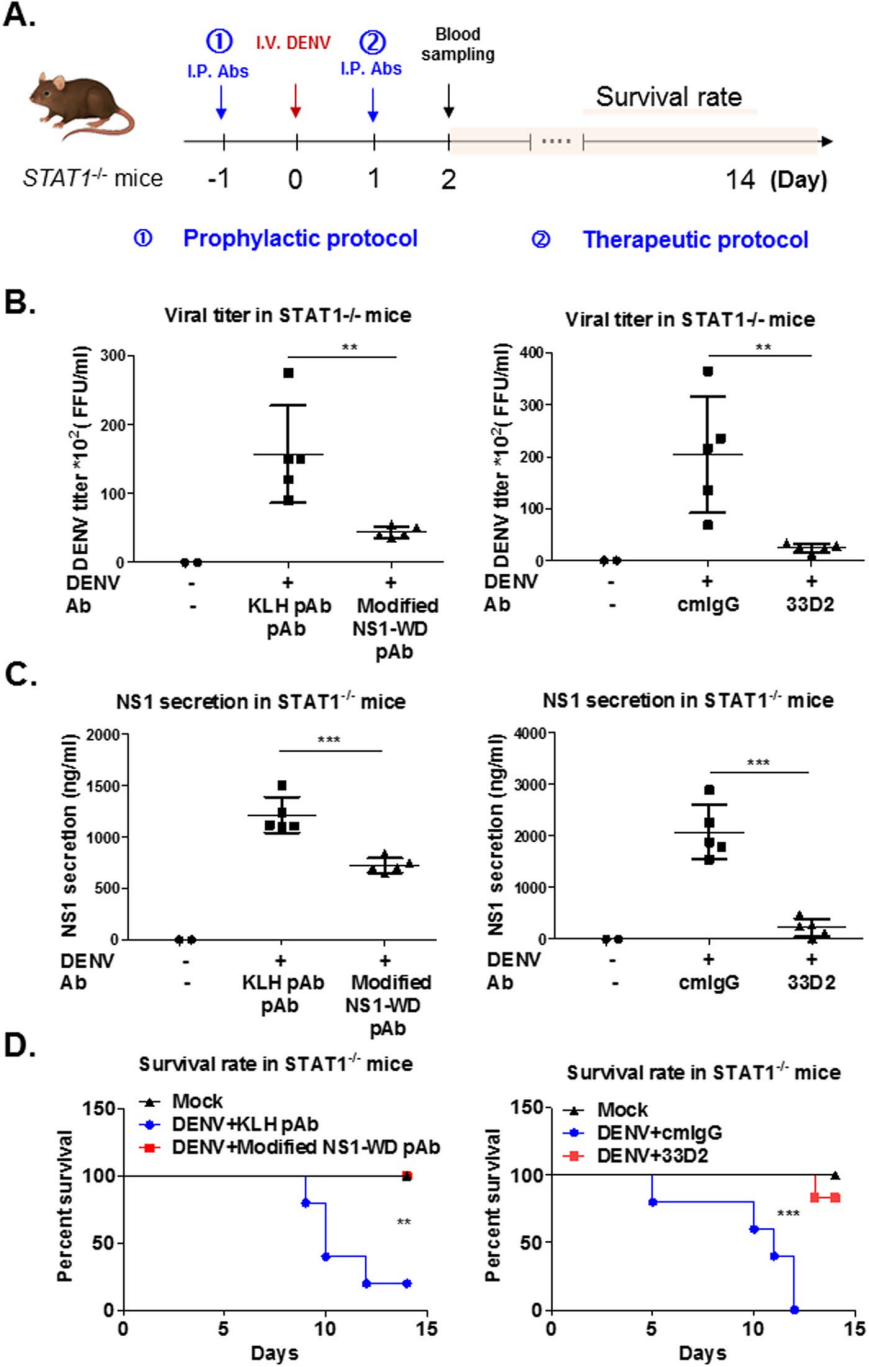
Protection against lethal DENV 2 challenge in *STAT1* ^−/−^ mice by prophylactic or therapeutic treatment with Abs against modified NS1-WD peptide. (**A**) The schematic model for the prophylactic and therapeutic treatment of mice. For the prophylactic protocol, PBS (Mock, N = 2), anti-KLH pAb (100 μg/mouse, N = 5), or anti-modified NS1-WD pAb (100 μg/mouse, N = 5) were injected intraperitoneally (i.p.) one day before virus challenge. For the therapeutic protocol, PBS (Mock, N = 2), cmIgG (100 μg/mouse, N = 5), or mAb 33D2 (100 μg/mouse, N = 5) were injected i.p. one day after virus challenge. 4*10^7^ PFU/mouse DENV 2 (454009 A) or C6/36 control medium were inoculated intravenously (i.v.) into *STAT1* ^−/−^ mice. (**B**) Viremia in *STAT1* ^−/−^ mice at 2 days post infection was analyzed by FFA as described in the Methods. (Left panel represents prophylactic model; right panel represents therapeutic model.) (**C**) Sera of *STAT1* ^−/−^ mice were collected at 2 days post infection, and the sNS1 levels in sera were analyzed by NS1 quantitative ELISA. (Left panel represents prophylactic model; right panel represents therapeutic model.) (**D**). Survival rate of mice was monitored for 14 days and analyzed by Kaplan-Meier survival curves. P values represent the comparison between anti-modified NS1-WD pAb and control anti-KLH pAb or cmIgG and mAb 33D2-treated mice *P < 0.05, **P < 0.01, ***P < 0.001, ns indicates no significance.

Because immunodeficient mice are more susceptible to DENV infection^38–40^, we further established a lethal infection mouse model in *STAT1*-deficient mice (*STAT1* ^−/−^ mice) to test the prophylactic and therapeutic effects of these modified NS1-WD-peptide specific Abs against lethal DENV 2 infection (Fig. 6). We demonstrated that the non-mouse adapted DENV 2 strain 454009 A can actively replicate in *STAT1* ^−/−^ mice. Viremia in the sera of these mice could reach 10^3^–10^4^ PFU/ml at 2–3 days post infection. In addition, mice died within two weeks after DENV challenge. Administration of Abs against modified NS1-WD peptide 24 h before lethal DENV infection could reduce viremia, NS1 secretion and lethality in *STAT1* ^−/−^ mice. Furthermore, transfer of mAb 33D2 24 h after lethal DENV infection demonstrated therapeutic efficacy against DENV-induced viremia and mortality (Fig. 6B–D).

### Detection of Abs that can recognize the modified NS1-WD peptide in dengue patients

**Figure 7:**
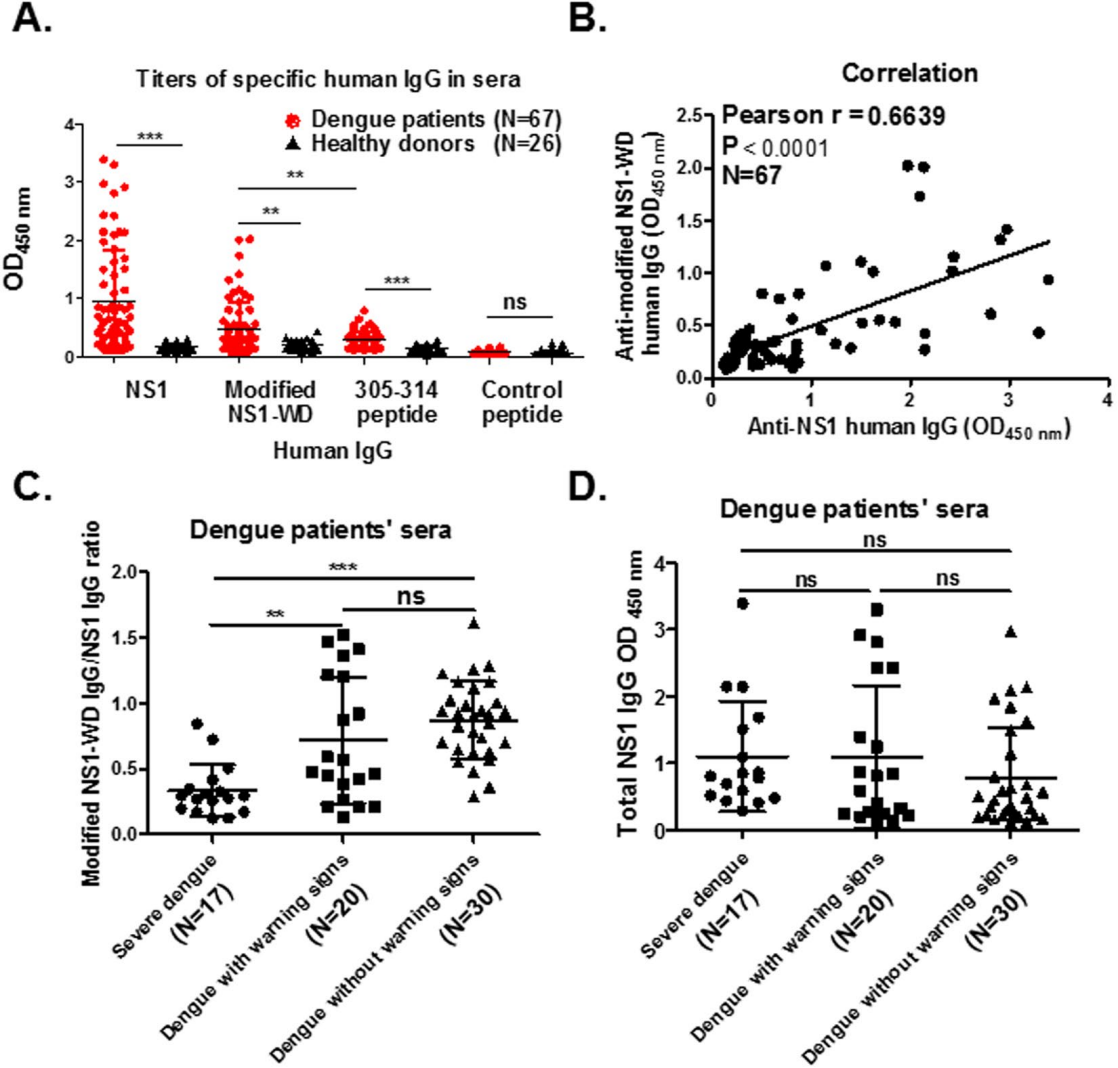
Antibodies bind to modified NS1-WD peptide in dengue patients. **(A)** The OD of antibodies against NS1, modified NS1-WD peptide-conjugated BSA, C terminal NS1 (a.a. 305–314)-conjugated BSA and control peptide (Nonspecific peptide)-conjugated BSA in the sera of dengue patients (N = 67), as well as healthy donors (N = 26), was determined by ELISA. **(B)** The correlation of the OD of anti-NS1 (x-axis) and anti-modified NS1-WD peptide (y-axis) antibodies in the sera of dengue patients (N = 67). **(C)** The OD ratio of Abs binding to modified NS1-WD peptide to total NS1 Abs was significantly reduced in severe dengue patients’ sera (N = 17) compared to dengue with (N = 20) or without (N = 30) warning signs. *p < 0.05, **p < 0.01, ***p < 0.001. **(D)** The comparison of the OD of total NS1 Abs in dengue patients with different disease severity.

To further investigate whether Abs recognizing the modified NS1-WD peptide are produced naturally in dengue infection, we collected sera from 67 cases of acute dengue patients and 26 cases of normal healthy individuals. We first screened for Abs binding to different peptides in these sera. Abs binding to the modified NS1-WD peptide and C-terminal NS1 peptide (a.a.305–314), but not control peptide, were increased in dengue patients’ sera compared to normal sera. Moreover, the mean optical density (OD) at 450 nm of Abs binding to modified NS1-WD peptide was higher compared to that of Abs binding to C-terminal NS1 peptide (a.a.305–314) (Fig. 7A). In addition, there was a positive correlation between the OD of Abs binding to modified NS1-WD and the OD of Abs binding to full-length NS1(Pearson r = 0.6639, p value < 0.0001) (Fig. 7B). Subsequently, we classified these dengue patients into different degrees of severity according to the suggested guidelines of dengue severity from the World Health Organization. We found that the OD ratios of Abs binding to modified NS1-WD peptide to total anti-NS1 Abs were significantly reduced in severe dengue patients’ sera compared to dengue with or without warning signs (p value < 0.01 and p value < 0.001, respectively) (Fig. 7C). However, no significant difference in the OD of total anti-NS1 Abs among dengue patients with different disease severity was found (Fig. 7D). Collectively, these results suggest that Abs recognizing the modified NS1-WD peptide are naturally produced in dengue patients, which may provide protective effects against DENV disease severity.

## Discussion

The present study offers a novel alternative to current dengue vaccine design. The development of a safe and efficacious dengue vaccine has been challenging because of the necessity of protecting against all four serotypes of DENV to prevent the risk of antibody-dependent enhancement (ADE)^41, 42^. According to the ADE hypothesis, Abs generated from a previous DENV infection are non-neutralizing but enhance a secondary heterotypic infection, which may contribute to the development of DHF/DSS. Indeed, non-neutralizing or sub-neutralizing Abs against DENV E enhance viral entry and infection, as has been shown not only *in vitro* but also *in viv*o^43–45^. Therefore, much effort in DENV vaccine development has been focused on how to generate equally effective Ab responses against all four serotypes of DENV. So far, a live attenuated chimeric tetravalent DENV vaccine has been licensed in several countries. However, this vaccine renders only partial protection against DENV infection and the incidence of hospitalized dengue cases among vaccinated children less than 9 years of age was elevated^46–48^. These shortcomings have prompted us to develop an NS1-based vaccine as an auxiliary approach to prevent dengue disease. In this study, we showed that both active immunization of mice with modified NS1-WD peptide and passive transfer of NS1 mAb 33D2 could provide broad-spectrum protection against different serotypes of DENV. These results suggest that the modified NS1-WD peptide may be an alternative DENV vaccine candidate.

The NS1 protein is a highly conserved glycoprotein among the four serotypes of DENV. Because NS1 is not a virion-associated protein, there is no risk of ADE. Although previous studies have shown that full-length DENV NS1-immune sera of mice protect mice against even heterologous serotypes of lethal DENV infection^25^, the potentially pathogenic effects of molecular mimicry between specific NS1 regions and host proteins have not been investigated. To avoid the potential problem of NS1-induced cross-reactive Abs, different strategies have been used previously. Since most of the cross-reactive Abs recognized the C-terminus (a.a. 271–352) of NS1, C-terminal region deleted or modified NS1 constructs have been generated. Both of these modifications have been shown to be able to reduce cross-reactivity with human platelets and endothelial cells. Furthermore, active immunization with these modified NS1 proteins provided notable protection against DENV-infection and DENV-induced hemorrhage in mice with minimal side effects^27^. The present study builds on these findings and, most importantly, eliminates the potentially harmful LYRIC sequence homology within the NS1-WD disordered loop^34^. To minimize the risk of inducing cross-reactive Abs, we analyzed two mAbs that can recognize the NS1-WD disordered loop. mAb 19–5 recognized an epitope containing the KXWG motif as previously reported^34^. mAb 33D2 recognized an epitope containing no KXWG motif (Fig. 2). After comparing the epitopes recognized by these two mAbs, we then redesigned the sequence and synthesized the modified NS1-WD peptide to immunize mice. We found that modified NS1-WD peptide immune sera retained the ability to recognize all four serotypes of DENV-infected cells but had reduced cross-reactivity to endothelial cells. Since there were only 11 amino acids in the modified NS1-WD peptide, we do not anticipate problems with secondary structure. In addition, this modified NS1 peptide-based dengue vaccine not only has the benefit of reducing the risk of ADE and autoimmunity but also minimizes the cost of vaccine production.

The protection against DENV infection afforded by modified NS1-WD peptide immunization may be mediated by several mechanisms. Modified NS1-WD-specific Abs may trigger complement-mediated lysis of DENV-infected cells or antibody-dependent cell-mediated cytotoxicity by recognition of surface NS1. This mechanism is supported by our *in vitro* results, shown in Fig. 4, which showed that modified NS1-WD Abs could attenuate DENV propagation in the presence of complement. Furthermore, we also demonstrated that mice immunized with modified NS1-WD peptide could facilitate complement c1q fixation around DENV-infected cells in Fig. 5E. Most interestingly, based on the Zika NS1 structure obtained by George Gao, a conserved aromatic amino acid-rich region (consisting of Y122, F123, and V124) within the NS1 wing domain may form a hydrophobic spike that associates with membrane(s)^49^. Because the epitope recognized by mAb 33D2 is from a.a.112 to 121, which is very close to the hydrophobic spike, the region recognized by mAb 33D2 is likely very close to the membrane. Due to the membrane-proximity of the binding site recognized by mAb 33D2 and pAb against modified NS1 WD peptide, complement activation induced by both antibodies could effectively lyse DENV-infected cells^50^.

In addition to complement-mediated protection against surface-expressed NS1, modified NS1-WD Abs may inhibit the pathogenic effects of secreted NS1 as well. Previously, we have shown that secreted NS1 can interact with prothrombin/thrombin and perturb the function of coagulation^22^. Recently, secreted NS1 has been shown to bind to endothelial cells directly or through the induction of inflammatory cytokine production, leading to the loss of endothelial integrity and vascular leakage^26, 51^. Furthermore, anti-NS1 Abs can counteract endothelial hyperpermeability and reduce mortality in DENV-infected mice^25^. As shown in Supplementary Fig. 4, there was a peak of NS1 secretion in mice 12 h after live but not UV-inactivated DENV challenge, indicating NS1 was *de novo* synthesized in the disease mouse model we used in this study. In addition, DENV-induced vascular leakage can be observed in lungs and skin of mice, which can be rescued in the presence of mAb 33D2 in this mouse model. Thus, NS1-WD specific mAb 33D2 or modified NS1-WD Abs induced by immunization may inhibit secreted NS1-elicited vascular leakage as well. Importantly, injection of modified NS1-WD pAb into *STAT1* ^−/−^ mice before DENV challenge to mimic modified NS1-WD peptide immunization in these mice reduced viremia and NS1 secretion and provided protection against DENV-induced mortality. Similar protective effects were found when mAb 33D2 was giving after DENV infection in *STAT1* ^−/−^ mice as therapeutic treatment. These results suggest that modified NS1-WD peptide may not only be a vaccine candidate against DENV infection but also that its antibodies can provide therapeutic effects against DENV infection.

To investigate whether Abs that can recognize modified NS1-WD peptide can be induced during DENV infection in patients, we analyzed the sera of dengue patients with different disease severity. Our results demonstrated that Abs that recognize modified NS1-WD peptide were indeed detectable in dengue patients’ sera. Moreover, disease severity inversely correlated with the ratio of Abs that recognized modified NS1-WD peptide to total anti-NS1 Abs. Patients with lower ratios tended to show more severe symptoms. Even though future prospective studies are required to know whether the ratio of Abs that recognize modified NS1-WD to total NS1Abs can be a predictor of disease severity, our current results suggest that it is likely that Abs recognized modified NS1-WD peptide may provide protection against severe dengue symptoms in patients.

In summary, we demonstrated in this study that both passive transfer of mAb 33D2 as well as active immunization with the modified DENV NS1-WD peptide can protect mice against DENV-induced hemorrhage, coagulopathy, vascular leakage, viremia, NS1 secretion and mortality, suggesting that they can be a therapeutic or preventative option against dengue disease.

## Methods

### Viral Stocks and cell lines

Human hepatoma cell line HuH-7, Baby hamster kidney cell line BHK-21 and *Aedes albopictus* cell line C6/36, purchased from the Japanese Collection of Research Bioresources (Japan) and the American Type Culture Collection (ATCC, Manassas, Virginia), were maintained in Dulbecco’s Modified Eagle’s Medium (DMEM) supplemented with 10% heat-inactivated fetal bovine serum (FBS, HyClone, Logan, UT). Human umbilical vein endothelial cells (HUVECs) were purchased from the Bioresource Collection and Research Center (BCRC) of Taiwan and maintained in endothelial Basal Medium-2 (EMB-2, Lonza, Walkersville, MD) supplemented with 10% FBS, and SingleQuots™ Kit (Lonza, Walkersville, MD). All cells were cultured at 37 °C in a 5% CO2 atmosphere, except for C6/36 which were cultured at 28 °C. Four serotypes of dengue viruses, DENV 1 (local Taiwan strain 8700828), DENV 2 (16681 and local Taiwan strain 454009 A), DENV 3 (local Taiwan strain 8700829), and DENV 4 (local Taiwan strain 59201818) were propagated in C6/36 cells as previously described^52^. To prepare high titers of DENV, cell-free supernatants were concentrated by Macrosep® Advance Centrifugal Devices (molecular weight cutoff of 30 kDa; Pall Corp., Port Washington, NY) at 6000 × g at 4 °C and stored below −70 °C until use.

### Virus titration and fluorescent focus assay (FFA)

For virus titration, the fluorescent focus assay was used to determine the virus titer according to a previous study^53^. In brief, supernatants containing infectious virus were collected and stored below −70 °C until use. Supernatant was serially diluted and incubated with BHK-21 cells for 2 h at 37 °C. The monolayers were then overlaid with DMEM containing 2% FBS and 1% methylcellulose and incubated at 37 °C for 2–3 days. Virus foci were stained with anti-NS1 antibody (mAb 33D2) followed by Alexa 488-conjugated goat anti-mouse IgG (Invitrogen, Carlsbad, CA) and visualized with a fluorescence microscope (Leica Geosystems AG, St. Gallen, Switzerland).

### Mice and generation of mAbs

The C3H/HeN and BALB/c mice used in this study were obtained from the Laboratory Animal Center of National Cheng Kung University (NCKU), and the *STAT1* ^−/−^ C57BL/6 mice were obtained from Dr. Chien-Kuo Lee (National Taiwan University College of Medicine), which were maintained on standard laboratory food and water in the Laboratory Animal Center of NCKU. Housing and experimental use of the mice were performed in accordance with the Experimental Animal Committee of NCKU. The experiments were approved by the Institutional Animal Care and Use Committee of NCKU. To generate mouse mAb, insect cell-derived DENV 2 NS1 immunized mice were used and hybridomas were generated according to the hybridoma technique as described in the supplementary information^54^.

### Enzyme-linked immunosorbent assay (ELISA)

For indirect ELISA, 50 μl of NS1, bovine serum albumin (BSA), peptides-conjugated BSA or antibody (2 μg/ml) in PBS (pH 7.3) was coated onto 96-well ELISA plates at 4 °C overnight. After blocking for 1 h with 1% BSA in PBS, Abs, or mice or human sera (1:50 dilution) were incubated on wells at 37 °C for 1 h. Next, horseradish peroxidase (HRP)-conjugated goat anti-rabbit, anti-mouse IgG (Leadgene Biomedical, Taiwan), anti-human IgG (Jackson ImmunoResearch Laboratories, West Grove, PA) or mouse anti-M13 (Zymed Laboratories, California, USA) secondary Abs (1:10,000 dilution) were incubated on wells at 37 °C for another hour.

For competitive ELISA, Abs (0.1 μg/ml) were pre-incubated with different doses of control peptide (NYRATATEPHC) or LYRIC peptide (NTNGKDWGRSC), as indicated, for 1 h at 37 °C. Subsequently, Ab-peptide mixtures were incubated in NS1 (2 μg/ml)-coated plates for another 30 min, followed by washing with PBST (PBS containing 0.01% Tween 20) and incubated with HRP-conjugated goat anti-mouse IgG for another 30 min.

Subsequently, plates were washed with PBST, followed by color development and visualization using tetramethylbenzidine (TMB, Clinical Science Products, Mansfield, MA) as the substrate. The absorbance was read after adding stop solution (2 N H_2_SO_4_) at OD_450_ nm by a VersaMax microplate reader (Molecular Devices, Sunnyvale, CA).

### Immunoprecipitation and Immunoblotting

For immunoprecipitation assays, HUVECs (1 × 10^7^) were lysed with lysis buffer (20 mM Tris-HCl, pH 8.0, 137 mM NaCl, 2 mM EDTA, 1% Nonidet P-40) supplemented with protease inhibitors (Roche Complete protease inhibitor cocktail, Roche Diagnostics Ltd, Mannheim, Germany) and kept on ice for 30 min. The supernatant was prepared by centrifugation at 10,000 × g for 15 min at 4 °C, and pre-cleaned by protein G-coated magnetic beads (Dynabeads, Invitrogen, Carlsbad, CA) for 1 h at 4 °C. After pre-cleaning, lysate was incubated with additional Ab (2 μg/ml)-saturated protein G-coated magnetic beads at 4 °C overnight. After washing, the immunocomplex was fractionated in 8% SDS-PAGE and immunoblotted with anti-LYRIC Ab (GTX100587, GeneTex, Inc, Irvine, CA).

### Epitope mapping using phage-display random peptide library

To determine the epitopes recognized by mAbs, we used a phage-display random peptide library kit (PhD 12-mer; New England Biolabs, Ipswich, MA) according to the manufacturer’s instructions. In brief, mAbs (10 nM) were captured by protein G-magnetic beads (Dynabeads; Invitrogen, Carlsbad, CA) for 30 min, followed by washing with 1 ml Tris-buffered saline contained 0.5% Tween 20 (TBST). Phages (2 × 10^11^) from the original library were incubated with mAb complexes for 30 min, followed by washing with 1 ml TBST 10 times. Negative selection with normal mouse IgG was performed at the second and third rounds of panning. Unbound phages from negative selection were further incubated with mAb and washed as described above. Bound phages were eluted with glycine buffer (pH 2.2) and neutralized by 1 M Tris-HCl (pH 9.0) immediately, followed by amplification for subsequent rounds of panning. After four rounds of panning, the specific binding of positive single phage clones against mAbs were confirmed by ELISA, and their DNA sequences were analyzed using extracted ssDNA following the manufacturer’s suggestions.

### Complement-mediated cytolysis and LDH release assay

To analyze complement-dependent cytolysis of infected cells, HUVECs (8 × 10^3^) were infected with DENV (multiplicity of infection = 10) for 48 h. Cells were washed with PBS and then incubated with 56 °C heat-inactivated Abs (50 μg/ml) for 1 h at 4 °C. After washing with PBS, cells were incubated with Low-Tox-M rabbit complement (1:20 dilution) (Cedarlane Laboratories Ltd, Ontario, Canada) containing 2% FBS EMB-2 medium for another 4 h. Then, 50 μl supernatants were collected and mixed with 50 μl CytoTox 96® substrate Reagent (Promega, Madison, Wis.) in each well. After 30 min incubation in the dark, 50 μl of stop solution were added into each well. Finally, the absorbance was read at 490 nm by a VersaMax microplate reader.

### Surface and intracellular protein staining

To visualize intracellular or surface antigens on cells, immunofluorescence staining and flow cytometry were used. For indirect immunofluorescence (IIF), cells were fixed with 4% paraformaldehyde for 20 min. After penetration with or without 0.5% Triton X-100 for intracellular or surface staining, respectively, cells were stained with primary antibody at 4 °C overnight, followed by staining with Alexa 488-conjugated goat anti-rabbit or anti-mouse IgG secondary Abs and nuclear-staining, Hoechst 33342 (2 µg/ml) (Invitrogen, Carlsbad, CA). Bound Alexa-conjugated Abs and Hoechst were detected by Multi-photon Confocal Microscope with an oil immersion lens (×60, NA: 1.35). For flow cytometry analysis, cells were detached with PBS containing 4 mM EDTA and washed with PBS. After primary antibody staining, Alexa 488-conjugated secondary Abs were used. Detection and analysis were performed using a FACS Calibur Flow Cytometer (Becton Dickinson, Mansfield, MA). The quantification was analyzed using WinMDI 2.9 software.

### Disease and lethal infection models in mice

C3H/HeN wildtype mice were used in a DENV-induced disease murine model, which was modified from previous publication^37^. For active immunization, synthetic modified NS1-WD peptide-conjugated KLH (ChinaPeptides, Shanghai, China), or control protein (KLH) was emulsified with Complete or Incomplete Freund’s Adjuvant (CFA, IFA). 6-week-old mice were intraperitoneally immunized with CFA-emulsion for two weeks and boosted with IFA-emulsion every week three times, until there were sufficient antibodies against NS1 in the sera. Three days following the last immunization, the mice were challenged intradermally with concentrated DENV (2*10^8^ PFU/mouse) or concentrated C6/36 medium as a control at four sites on the upper back. For passive immunization, one injection of Abs (100 μg/mouse) was given intraperitoneally after 24 h of DENV inoculation. Three days after challenge, the bleeding time from the tail vein was determined according to a previous publication and mice were sacrificed and their skins were removed to observe hemorrhages and perform immunohistochemistry (IHC) or Hematoxylin and Eosin (H&E) staining. The degree of hemorrhage was digitally quantified by ImageJ software^55^.

5- to 6-week-old *STAT1* ^−/−^ mice were used in a lethal infection murine model. A DENV 2 lethal strain (454009 A) (4*10^7^ PFU/mouse) or C6/36 control medium were inoculated intravenously (i.v.) into *STAT1* ^−/−^ mice. For the therapeutic model, one injection of Abs (100 μg/mouse) was injected intraperitoneally after one day of DENV challenge. For the prophylactic model, purified pAbs (100 μg/mouse) from sera of immunized mice were injected intraperitoneally one day before DENV challenge. Sera of mice were collected at 2 days post-infection to determine viremia and NS1 secretion by FFA and quantitative NS1 ELISA. Body weight and survival rates of mice were monitored for 14 days.

### Hematoxylin and Eosin staining (H&E stain) and Immunohistochemistry (IHC)

Fresh mice skins were fixed with 4% formalin and embedded with paraffin. For histopathology analysis, tissue sections were stained with H&E. For immunohistochemistry staining, the slides were blocked following the manufacturer’s instructions (BIOTnA biotech) and incubated with anti-NS3 Ab (GTX124252, GeneTex, Inc, Irvine, CA) or anti-c1q Ab (ab 71089, Abcam, Cambridge, MA) at 4 °C overnight. After washing with PBS, the slides were incubated with HRP-labeled secondary antibody for 30 min at room temperature and then loaded onto 3,3′-diaminobenzidine (DAB) or HRP green mixed reagent for 1–5 min. Hematoxylin counterstain was applied for 2 min. The quantification of DAB staining was determined by ImageJ software.

### Human sera collection

In this study, sera from 67 confirmed dengue patients were collected from National Cheng Kung University Hospital (NCKUH), at the acute stage (Day 0–7 after onset of illness) of disease during a DENV outbreak in Tainan, Taiwan, 2015. The characteristics of these clinical samples are shown in Supplementary Table 1. The laboratory standard set forth by the Taiwan Centers for Disease Control was followed for dengue infection diagnosis. The severity of dengue cases was classified as severe dengue, dengue with warning signs, or dengue without warning signs, according to the latest national guidelines from the World Health Organization^4^. In addition, 26 healthy donors’ sera were included as negative controls. All the sera collections were performed in accordance with the relevant guidelines and regulations approved by the institutional review board of NCKUH (IRB #A-BR-101–140) and the informed consent was obtained from all participants and/or their legal guardians.

### Statistical analysis

All statistical analyses were performed using Prism software (GraphPad Software Inc., CA). The results were analyzed using the unpaired Student’s *t*-test or one-way ANOVA test to compare two independent groups or more than two comparisons, respectively. For each result, all data are presented as the means ± S.D. from three independent experiments. The survival rate was analyzed by Log-rank Mantel-Cox test. *P < 0.05, **P < 0.01, ***P < 0.001, and ns indicates no significance for 95% two-tail confidence intervals.

## Electronic supplementary material

Supplementary information
