# Supplementary material for: Antibodies Against Modified NS1 Wing Domain Peptide Protect Against Dengue Virus Infection
Source: Sci Rep. 2017 Aug 1;7:6975. doi: 10.1038/s41598-017-07308-3 (PMC5539099; doi:10.1038/s41598-017-07308-3)
Supplement: Supplementary file 1 — Supplementary information [file 41598_2017_7308_MOESM1_ESM.doc]

**Title:**

**Antibodies Against Modified NS1 Wing Domain Peptide Protect Against Dengue Virus Infection**

**Authors:**

Yen-Chung Lai , Yung-Chun Chuang , Ching-Chuan Liu , Tzong-Shiann Ho ,Yee-Shin Lin , Robert Anderson, and Trai-Ming Yeh

**Supplementary Methods**

**Generation of mAbs**

To generate mouse mAbs, five to six-week-old BALB/c mice were immunized intraperitoneally with 50 μg insect cell-derived DENV 2 NS1 generated from drosophila (Biotech, San Diego, CA; a gift from Dr.Guey-Chuen Perng) by the general process of immunization. Hybridomas were generated according to the hybridoma technique.^1^ In brief, splenocytes were fused with mice myeloma FO cells and selected with modified selected-medium containing hypoxanthine–aminopterin–thymidine (GIBCO BRL Life Technologies, Invitrogen Corporation, CA,), 15% FBS (HyClone, Logan, UT) and 2.5% Hyboost (Leadgene Biomedical, Taiwan). Limiting dilution of the hybridomas was performed to obtain a single colony. Supernatants were collected and screened for antibodies against NS1 and NS1-WD peptide by ELISA. mAbs were purified from ascites of mice intraperitoneally injected with hybridomas by protein G-Sepharose (GE Healthcare), dialyzed against PBS (pH 7.3), and stored below -20°C.

**NS1 quantitative ELISA**

To analyze the concentration of NS1 in sera of mice, homemade NS1 sandwich ELISA was performed. In brief, 5 μg/ml anti-NS1 mAb 33D2 was coated onto 96-well plates at 4˚C overnight. After blocking for 1 h with 1% BSA in PBS, sera or plasma of mice (1:10 dilution) were co-incubated with 1 μg/ml biotin-conjugated anti-NS1 mAb 31B2 on wells at 37˚C for 1~2 h. Next, HRP-labeled streptavidin solution (1:200) (R&D Systems, Minneapolis, MN) was added into wells at 37˚C for 20 min. After washing with PBST (PBS contained 0.01% Tween 20) five times, color development and visualization with TMB was performed. The absorbance was read following the addition of stop solution (2N H_2_SO_4_) at OD_450_ nm by a VersaMax microplate reader.

**Skin hemorrhage quantification**

The degree of murine skin hemorrhage was image-processed by Photoshop® and digitally quantified by ImageJ software according to a published quantitativemethod.^2^ In brief, the samples of mice skin were collected and adjusted to the same image sizes. Then, the hemorrhagic areas were isolated and created as new images in Photoshop 6.0 (Adobe, San Jose, CA). To quantify the hemorrhagic portion, the processed images were loaded into ImageJ software, and converted into 16-bit images. After image type was set to black and white, the total hemorrhage volume was calculated and analyzed by Prism software.

***In vitro* permeability assay**

To analyze the permeability change of endothelial cells *in vitro*, transwell permeability assays were performed as previously described.^3^ Briefly, HUVECs (2x10^5^) were seeded on a transwell insert (0.4 μm; Corning B.V. Life Sciences, The Netherlands) until a monolayer was formed. The upper chambers were refilled with fresh medium containing 2% FBS with PBS, NS1 (20 μg/ml) with or without mAbs (10 μg/ml). After 6 h incubation, the media in the upper chambers were changed to 300 μl of serum-free media containing 1.25μl HRP-labeled streptavidin solution. The medium (20 μl) in the lower chamber was collected 10 min after adding streptavidin-HRP, and color development was performed by adding 100μl TMB. The absorbance was read following the addition of stop solution (2N H_2_SO_4_) at OD_450_ nm by a VersaMax microplate reader.

***In vivo* permeability assay**

To analyze the tissue permeability change in mice, the in vivo permeability assay was performed, according to a previous study.^4^ Briefly, 200 μl PBS containing 0.5% Evans blue was intravenously injected into mice tail veins for 30 mins. After euthanasia, the tissues were collected and extracted by formamide for 24 h. Evans Blue extravasation was detected and measured by absorbance of OD_600_ nm. The relative permeability was normalized to the tissue mass.

**Supplementary Table**


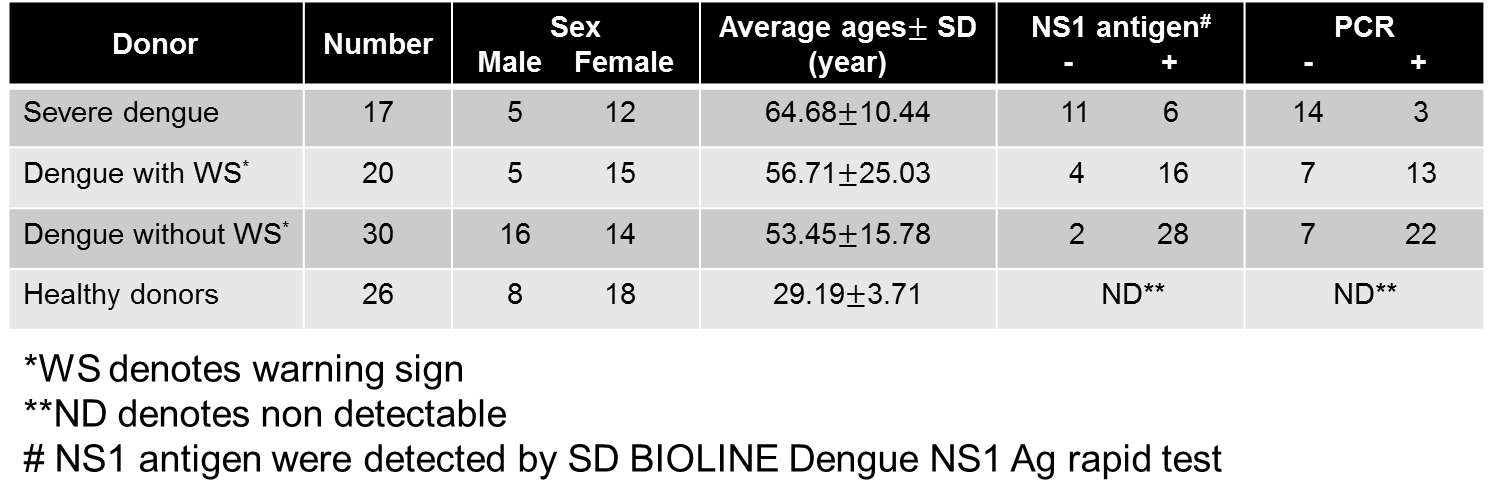


**Table 1. The characteristics of clinical samples and diagnostic criteria from the laboratory standard set forth by the Taiwan Centers for Disease Control.** The suspected dengue cases were confirmed by at least one of the following criteria: DENV RNA was detected by reverse transcription–polymerase chain reaction (RT-PCR)^5^; NS1 antigen was detected by NS1 strips; or DENV-specific immunoglobulin was detected by IgM/IgG strip test. Average ages are shown as the means ± SD.

**Supplementary Figures**


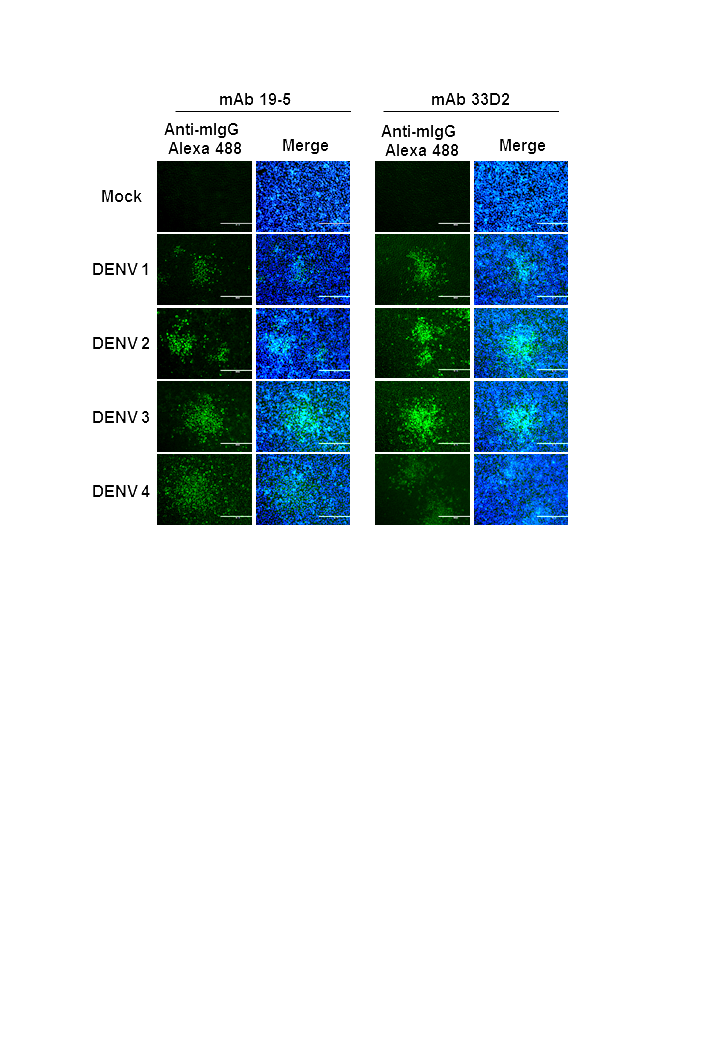
 **Fig. 1. Both mAb 19-5 and 33D2 can recognize four serotypes of DENV-infected cells.** BHK-21 cells were infected with four serotypes of DENVs (moi=2) or mock-infection for 48 h, followed by fixation and penetration. The staining of mAb 19-5 or 33D2 was detected with anti-mIgG Alexa 488 and analyzed by IIF. The images were taken by fluorescence microscopy at a magnification of 400x.

**
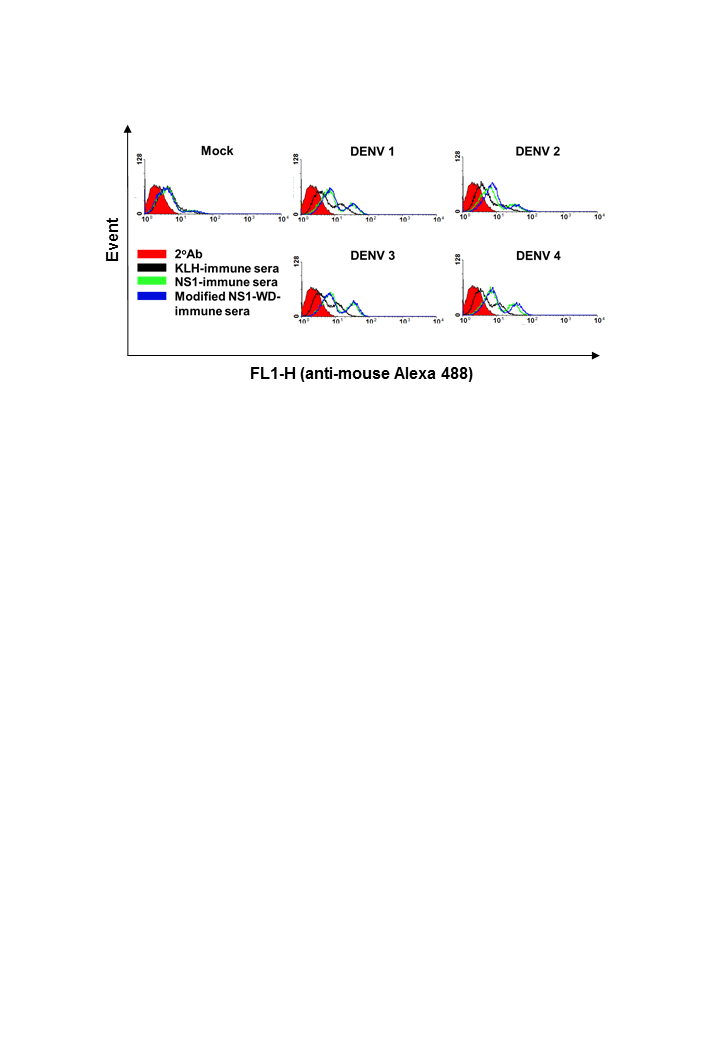
**

**Fig. 2. Modified NS1-WD immune sera of mice can bind to the surface of different serotypes of DENV-infected HuH-7 cells.** DENV 1-4 or mock-infected HuH-7 cells were stained with KLH, NS1, or modified NS1-WD peptide immune sera of mice (1:100 dilution) and analyzed by flow cytometry as described in the Methods.


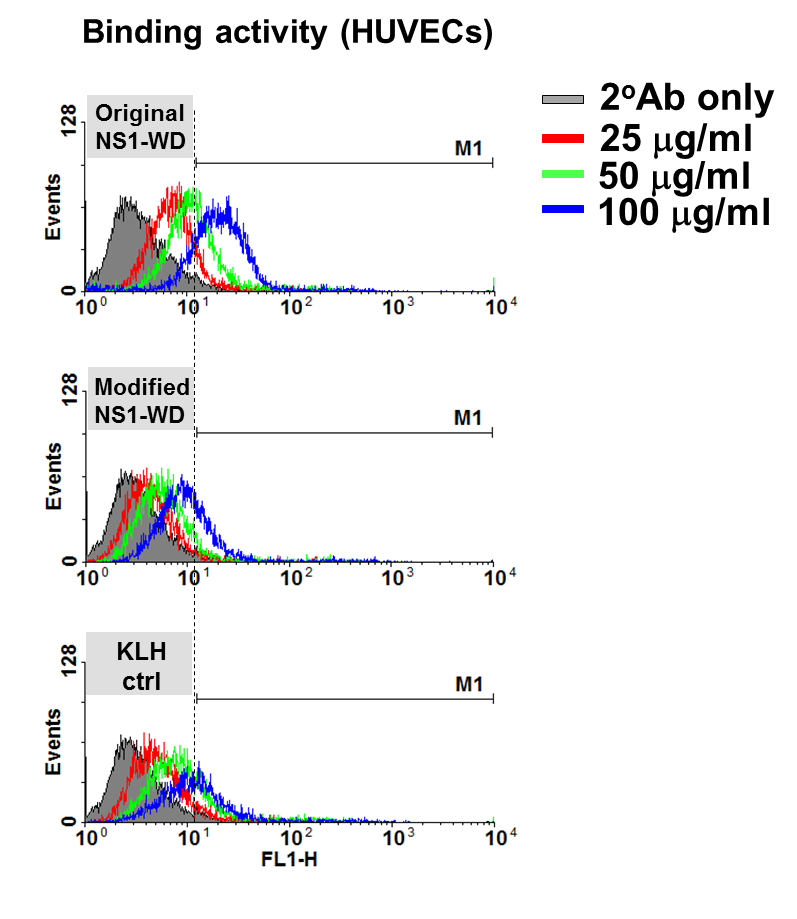


**Fig. 3. The binding of modified NS1-WD peptide-induced Abs to HUVECs is reduced.** Representative FACS graph of antibody binding activity to HUVECs was performed, similar to as same as **Fig. 2E**. The binding of different doses of purified pAbs from either original NS1-WD, modified NS1-WD peptide, or KLH immunized sera of mice to HUVECs was analyzed by flow cytometry.

**Fig. 4.** **The kinetic of NS1 secretion in sera of DENV 2-infected mice.** C3H/HeN mice (N=3) were inoculated with either DENV 2 or UV-inactivated DENV 2 (2*10^8^ PFU/mouse) intradermally at four sites on the upper back. Sera of mice were collected at the indicated time points and the NS1 secretion in mice was analyzed by NS1 quantitative ELISA.

**
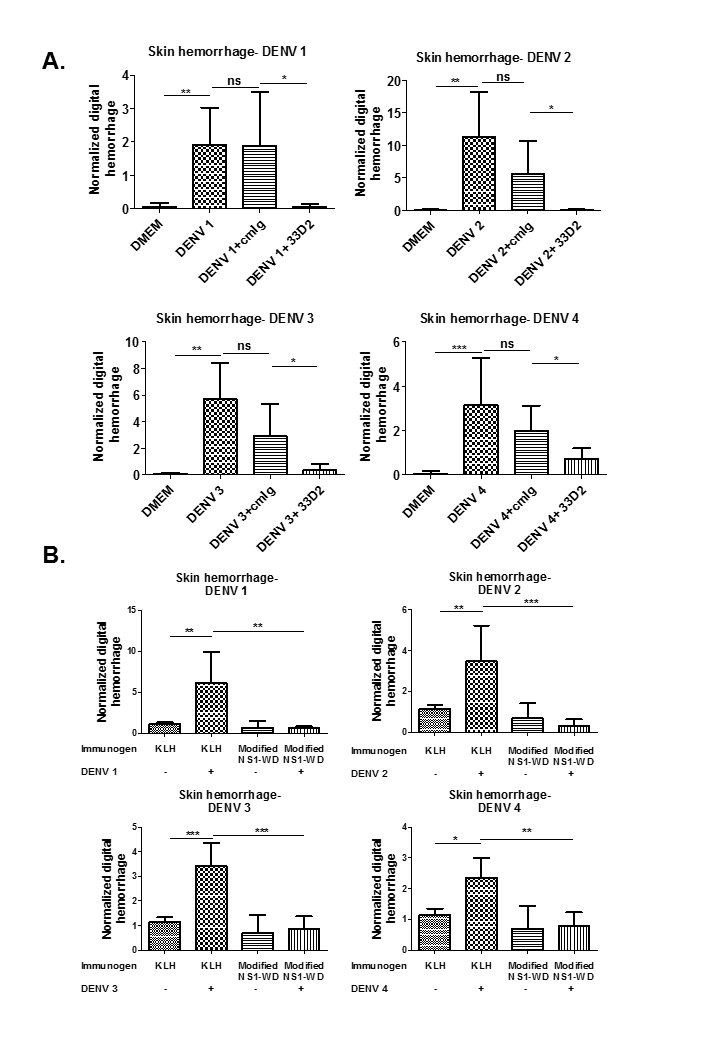
**

**Fig. 5. Passive transfer of mAb 33D2 attenuated all four serotypes of DENV-induced hemorrhage in mice.** DENVs (2*10^8^ PFU/mouse) or C6/36 control medium were inoculated intradermally (i.d.) into C3H/HeN mice three days before sacrifice. One day after virus challenge, PBS, cmIg, or mAb 33D2 were injected intraperitoneally (each N=5). Then, on day 3, mice were sacrificed, and the clinical score was quantified and determined as digital hemorrhage severity by ImageJ. *P<0.05, **P<0.01, ***P<0.001, ns indicates no significance.

**
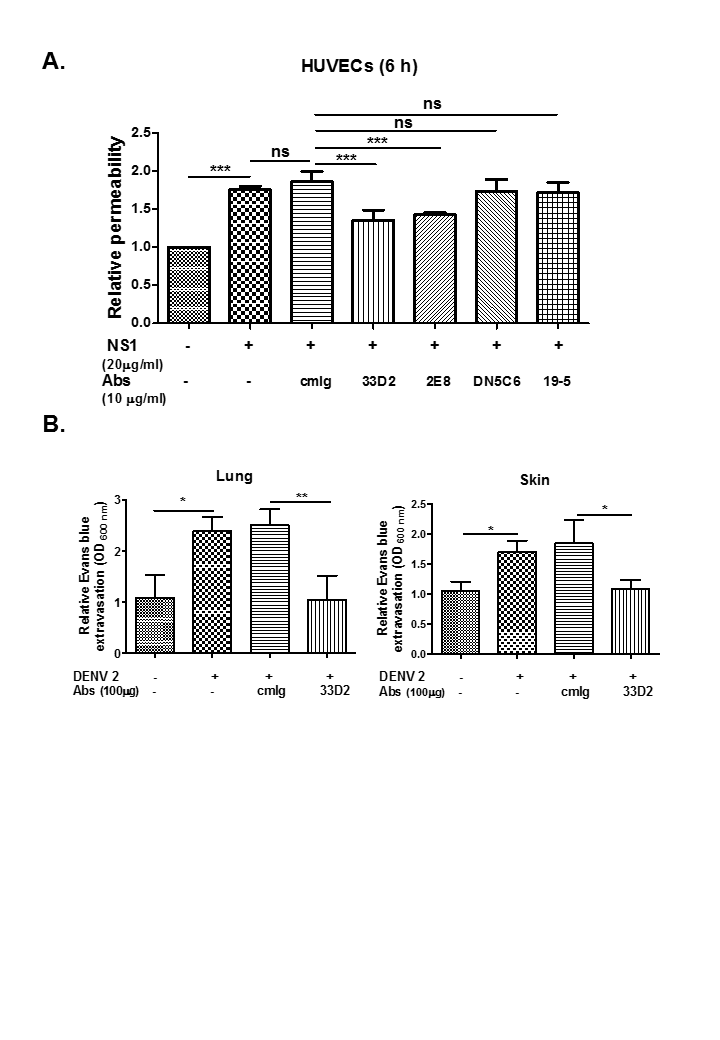
**

**Fig. 6. Anti-NS1 mAb 33D2 attenuated NS1- and DENV-induced vascular permeability both *in vitro* and *in vivo*.** (A) After 6 h treatment of NS1 with or without anti-NS1 mAbs (33D2, 2E8, DN5C6, and 19-5) or cmIg, the *in vitro* permeability was analyzed as described in the Supplementary Methods. Anti-NS1 Ab 2E8 served as a positive control. (B) C3H/HeN mice were challenged intradermally with concentrated DENV 2 (2*10^8^ PFU/mouse) or concentrated C6/36 medium as a control at four sites on the upper back. One injection of Abs (100 μg/ml) was given intraperitoneally 24 h after DENV inoculation. Three days after challenge, the vascular permeability change was analyzed by *in vivo* permeability assay as described in the Supplementary Methods. The relative vascular permeability was detected from mass-normalized lung and skin tissue at absorbance OD_600_ nm (each N=3) and quantified by ImageJ. All data are presented as the mean ± S.D. from at least three independent experiments.*P<0.05, **P<0.01, ***P<0.001, ns indicates no significance.

**
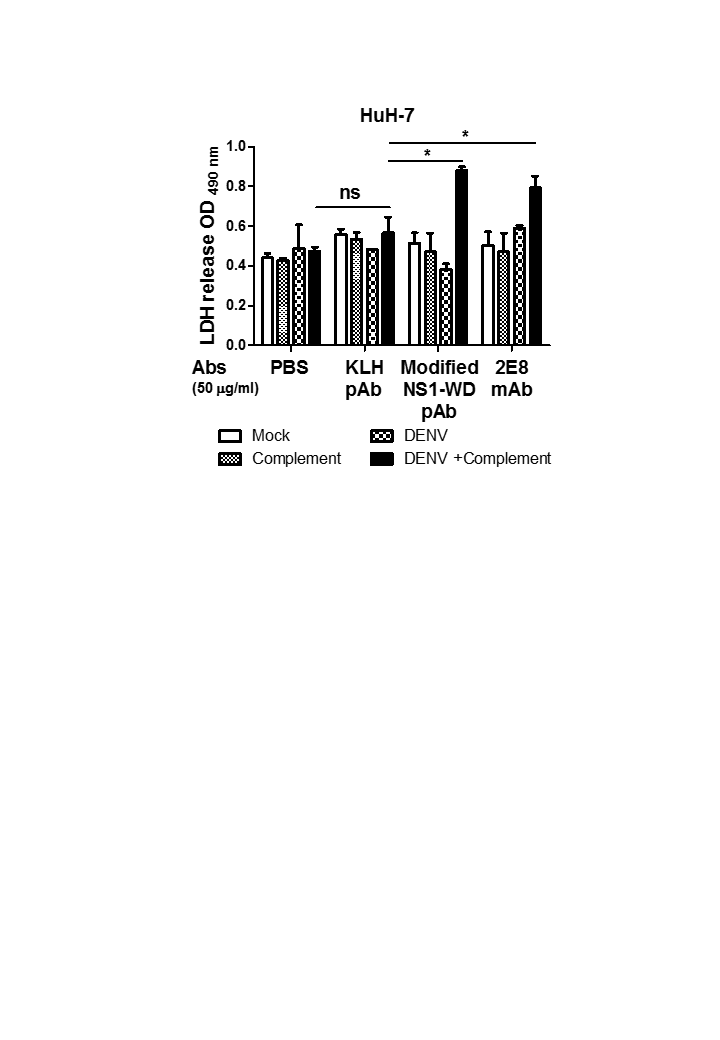
**

**Fig. 7. Antibodies against modified NS1-WD peptide induced-complement-
dependent cytolysis of DENV-infected HuH-7 cells.** HuH-7 cells were infected with DENV 2 (moi=5) or mock infection for 48 h. 48 h post infection, cells were incubated with either PBS, purified pAbs from KLH or modified NS1-WD-peptide immunized sera of mice (50 μg/ml), or anti-NS1 mAb 2E8 (50 μg/ml) for 1 h at 4°C and incubated with or without complement (1:20) for 4 h at 37°C. The release of lactate dehydrogenase (LDH) was analyzed as described in the Methods. Anti-NS1 Ab 2E8 served as a positive control. All data are presented as the mean ± S.D. from at least three independent experiments. *P<0.05, **P<0.01, ***P<0.001, ns indicates no significance.

**
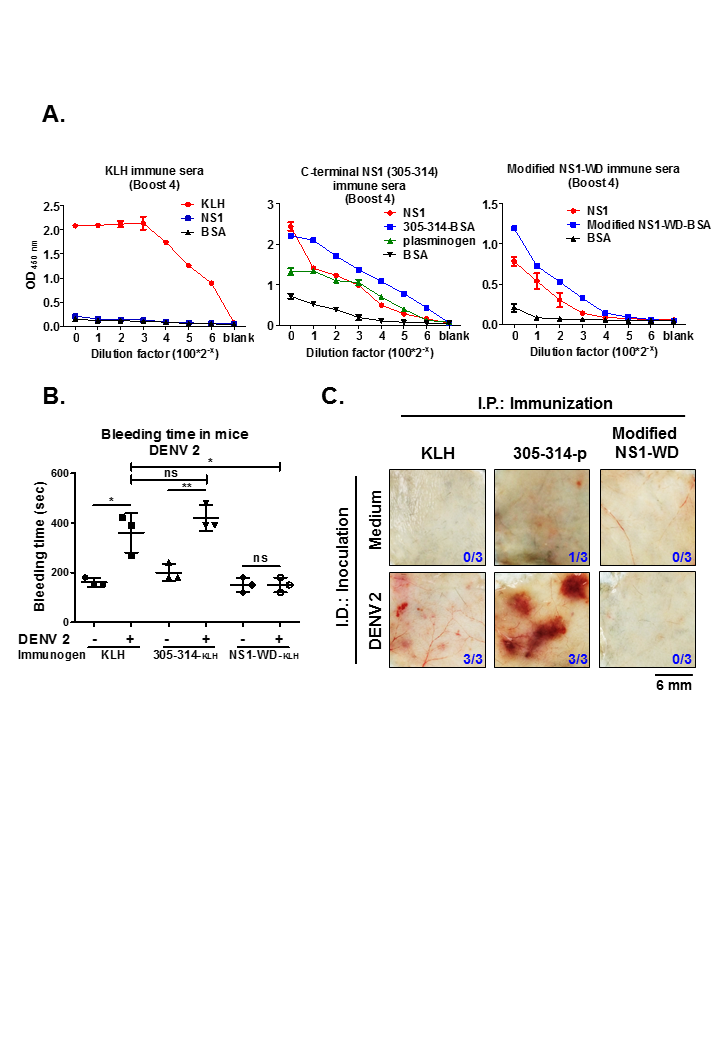
**

**Fig. 8. Active immunization with modified NS1-WD peptide but not C-terminal NS1 (305-314) can attenuate DENV 2-induced prolonged bleeding time and local hemorrhage in mice.** C3H/HeN mice (N=3) were intraperitoneally immunized with modified NS1-WD peptide-conjugated KLH, C-terminal NS1 (305-314) peptide-conjugated KLH or control protein (KLH) four times. Three days after the last immunization, the mice were inoculated with DENV 2 (2*10^8^ PFU/mouse) or C6/36 control medium intradermally at four sites on the upper back. (**A)** The antibody response against the indicated proteins was detected by indirect ELISA as described in the Methods. **(B)** The bleeding time from murine tail veins was determined as described in the Methods. **(C)** The skins from inoculated sites were collected to observe local hemorrhage.*P<0.05, **P<0.01, ***P<0.001, ns indicates no significance.


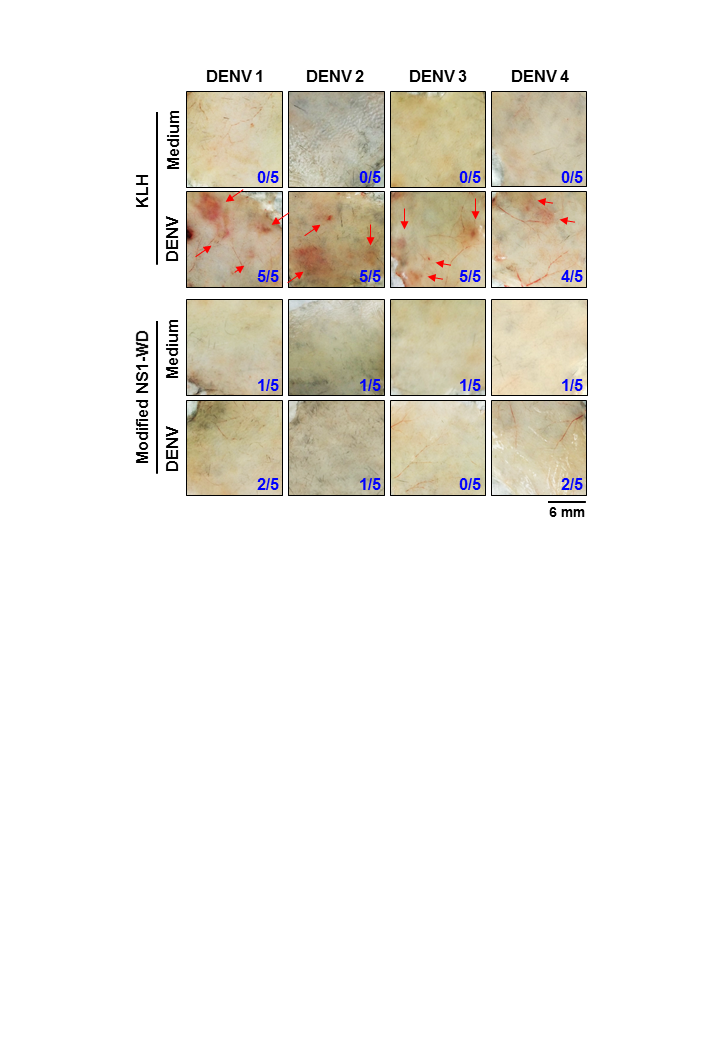
**Fig. 9.**  **Active immunization with modified NS1-WD peptide attenuated all four serotypes of DENV-induced hemorrhage in mice.** C3H/HeN mice (each N=5) were intraperitoneally immunized with modified NS1-WD peptide conjugated KLH or KLH alone four times. Three days after the last immunization, the mice were challenged with DENV (2*10^8^ PFU/mouse) or C6/36 control medium intradermally at four sites on the upper back. Then, on day 3, mice were sacrificed and the fresh skins from inoculated sites were removed to observe local hemorrhage. The number of mice with hemorrhage/total number of mice inoculated in each group are indicated. The scale bar indicates 6 mm.


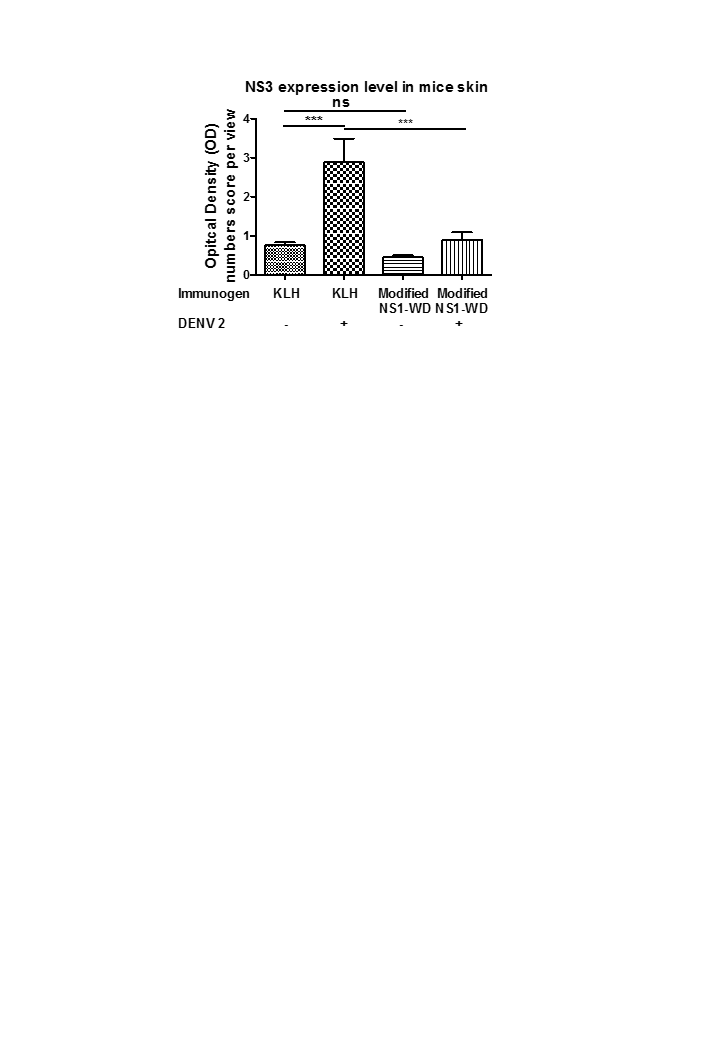


**Fig. 10. Quantification of NS3 expression in skin of mice.**

DENV NS3 expression was performed to analyze local DENV replication by IHC staining. The quantification of NS3 expression in six same-size images from three different tissues was performed with ImageJ software. *P<0.05, **P<0.01, ***P<0.001, ns indicates no significance.

**References:**

1 Nelson, P. N. *et al.* Monoclonal antibodies. *Mol Pathol* **53**, 111-117 (2000).

2 Tang, X. N., Berman, A. E., Swanson, R. A. & Yenari, M. A. Digitally quantifying cerebral hemorrhage using Photoshop and Image J. *J Neurosci Methods* **190**, 240-243, doi:10.1016/j.jneumeth.2010.05.004 (2010).

3 Chen, H. R. *et al.* Dengue Virus Nonstructural Protein 1 Induces Vascular Leakage through Macrophage Migration Inhibitory Factor and Autophagy. *PLoS Negl Trop Dis* **10**, e0004828, doi:10.1371/journal.pntd.0004828 (2016).

4 Radu, M. & Chernoff, J. An in vivo assay to test blood vessel permeability. *J Vis Exp*, e50062, doi:10.3791/50062 (2013).

5 Lanciotti, R. S., Calisher, C. H., Gubler, D. J., Chang, G. J. & Vorndam, A. V. Rapid detection and typing of dengue viruses from clinical samples by using reverse transcriptase-polymerase chain reaction. *J Clin Microbiol* **30**, 545-551 (1992).
